# Mums 4 Mums: structured telephone peer-support for women experiencing postnatal depression. Pilot and exploratory RCT of its clinical and cost effectiveness

**DOI:** 10.1186/1745-6215-12-88

**Published:** 2011-03-25

**Authors:** Isabela Caramlau, Jane Barlow, Sukhdev Sembi, Kirstie McKenzie-McHarg, Chris McCabe

**Affiliations:** 1 Warwick Medical School, University of Warwick, Gibbet Hill Road, Coventry, CV4 7AL, UK; 2 Department of Clinical Health Psychology, Warwick Hospital, Lakin Road, Warwick CV34 5BW, UK; 3 Institute of Health Sciences & Public Health Research, University of Leeds, Woodhouse Lane, Leeds, LS2 9JT, UK

## Abstract

**Background:**

Postnatal depression (PND) can be experienced by 13% of women who give birth, and such women often exhibit disabling symptoms, which can have a negative effect on the mother and infant relationship, with significant consequences in terms of the child's later capacity for affect regulation. Research has shown that providing support to mothers experiencing PND can help reduce their depressive symptoms and improve their coping strategies. The Mums4Mums study aims to evaluate the impact of telephone peer-support for women experiencing PND.

**Methods/Design:**

The study design adopts the MRC framework for the development and evaluation of complex interventions. Health visitors in Warwickshire and Coventry Primary Care Trusts are screening potential participants at the 8-week postnatal check using either the Edinburgh Postnatal Depression Scale (EPDS > = 10) or the three Whooley questions recommended by NICE (http://guidance.nice.org.uk/CG45). The Mums4Mums telephone support intervention is being delivered by trained peer-supporters over a period of four months. The primary outcome is depressive symptomatology as measured by the Edinburgh Postnatal Depression Scale. Secondary outcomes include mother-child interaction, dyadic adjustment, parenting sense of competence scale, and self-efficacy. Maternal perceptions of the telephone peer-support are being assessed using semi-structured interviews following the completion of the intervention.

**Discussion:**

The proposed study will develop current innovative work in peer-led support interventions and telecare by applying existing expertise to a new domain (i.e. PND), testing the feasibility of a peer-led telephone intervention for mothers living with PND, and developing the relationship between the lay and clinical communities. The intervention will potentially benefit a significant number of patients and support a future application for a larger study to undertake a full evaluation of the clinical and cost effectiveness of telephone based peer-support for PND.

**Trial registration:**

ISRCTN: ISRCTN91450073. The study has received a major funding grant from National Institute for Health Research (NIHR) (UK) - Research for Patient Benefit (RfPB) programme (ref: PB-PG-0407-13232).

## Background

Affective disorders following childbirth range from 'maternity blues' to postpartum psychosis, a serious condition requiring hospitalisation [1]. Along this spectrum postnatal depression (PND) is classified in DSM-IV as 'a depressive condition that often exhibits the disabling symptoms of dysphoria, emotional lability, insomnia, confusion, anxiety, guilt and suicidal ideation' [2]. A meta-analysis of 59 longitudinal and epidemiological studies showed a prevalence of PND in the region of 13%, ranging from 3 to 25% of women in the year following childbirth [3]. PND has been shown to affect both the mother and her baby, leading to mother-infant relationship difficulties [4] and long-term child behavioural [5-9], cognitive [10,11], and intellectual problems [12]; particularly for boys from disadvantaged backgrounds [8]. The treatment of PND is a public health priority, and recent UK National Institute for Health and Clinical Excellence guidance recommends that all women be screened for PND during the first eight weeks postnatally using the three Whooley questions to identify women experiencing difficulties [13]. It also suggests that women experiencing such problems should be offered support from health care professionals and voluntary organisations.

The aetiology of PND suggests the importance of a multitude of contributing factors such as life stresses, difficult infant behaviour, marital conflict, low maternal self-esteem and lack of social support [14,15]. Research has shown that factors such as the need to talk to someone who has experienced similar problems, lack of an intimate friend or confidante, the need for support without having to ask for it, and social isolation are all significant in the aetiology of PND (ibid). The use of 8 'listening visits' by specially trained health visitors has been identified as effective in supporting women experiencing PND [16]. However, the prevalence of PND is high and there is much unmet need, particularly for women who feel unable to admit to experiencing problems due to fears about being perceived as inadequate or the possibility of their baby being removed from the family. In addition, some women are not perceived to be experiencing sufficiently severe problems to justify additional support. This points to the potential value of developing peer-supporters. The NHS Expert Patient Report [17] recommends the development of lay-led self-management training programmes in order to make use of the knowledge and experience held by patients, and the White Paper "Our Health, Our Care, Our Say"[18] underlined the importance of assistive technology, with a strong emphasis on patient education and empowerment.

### The Evidence

A review of non-biological interventions for the treatment of PND identified four evaluations of the effectiveness of peer-support interventions [19]. The first three studies comprised evaluations of a post-partum support group targeting both depressed and non-depressed Canadian women [20], a Chinese evaluation of weekly support group meetings for depressed women only [21], and an Australian study of group-based support for postnatally 'distressed' women and their partners [22]. The studies suffered from serious theoretical limitations (such as the inclusion of both depressed and non-depressed women) and methodological weaknesses, rendering the results equivocal. The fourth study, however, comprised a Canadian telephone-based peer-support pilot RCT with women identified as being at high-risk of depression [14]. The findings showed significant group differences in depressive symptomatology at the 12-week assessment and support the provision of peer-support to women experiencing PND.

### Peer-support

Peer support has been defined as *"the giving of assistance and encouragement by an individual considered equal*" [23]. Another definition states "*that people who have like experiences can better relate and can consequently offer more authentic empathy and validation*". Individuals who have similar lived experiences can often offer practical advice and coping strategies of which health professionals may be unaware, and it is suggested that this non- professional approach is vital in helping people to re-connect with their community [24]. The most comprehensive definition of peer support within a healthcare concept is "*the provision of emotional, appraisal and informational assistance by a created social network member who possesses experiential knowledge of a specific behaviour or stressor and similar characteristics as the target population*" [23].

Research to identify the 'critical ingredients' of peer-support has identified three distinct factors: structure (program structure and environment), values (belief systems) and processes (peer-support, education/advocacy) [25]. The structural category defines how the support is constructed and its basic rules i.e. non-coerced, lay-led, safe, flexible informal setting, non-medical approach with no hierarchies. The value category refers to a set of belief systems, which include "*the peer principle" *(building an equal relationship with someone who has similar life experiences), "*the helper principle*" (the idea that helping someone else can be self-healing) and "*empowerment*" (discovering hope and the belief that recovery is possible, enabling someone to take personal responsibility for achieving their full potential). 'Process' refers to the way in which peer-support is delivered, such that it enables choice, encourages decision-making opportunities, and develops skills through knowledge and education, reciprocity, supportive mutual relationships, developing awareness, and a sense of community [25,26]. The underlying principle in terms of incorporating peer-support into health care is that new knowledge may be understood more effectively when it is communicated by a peer who has shared a common experience [27].

### Development of Mums4Mums: telephone peer-support for mums experiencing PND

The current proposal has adapted for use in the UK a telephone-based peer-support intervention shown to be effective in Canada [23], to pilot its use, and provide preliminary data on its effectiveness in reducing depressive symptoms amongst women experiencing PND.

The proposal builds on an exploratory study that examined a range of stakeholder's views about the need for, and potential acceptability of, a telephone-based peer-support intervention, their views about the potential impact of the intervention, and how it would fit into current practice. General Practitioners (GPs) (n = 6), health visitors (n = 7) and mothers who had recovered from PND (n = 10) were interviewed and the results indicated that stakeholders perceived a need for a telephone-based peer-support intervention for women currently experiencing PND in the UK. It was suggested that this would represent an additional resource for mothers, and that a telephone-based intervention would be acceptable due to its flexibility and use of non face-to-face contact.

The Mums4Mums telephone support intervention was piloted with women currently living with PND (n = 8). The pilot study was conducted to explore key elements of the telephone based peer-support intervention such as training, acceptability, and recruitment. In-depth interviews were again conducted, and the initial findings suggested that the Mums4Mums intervention was acceptable and potentially beneficial in supporting women with PND.

### Mums4Mums Feasibility Trial

The current study aims to test the feasibility of conducting a large-scale randomised controlled trial of a telephone based peer-support intervention to reduce depressive symptomology in women with PND.

### Research Objectives

The objectives of the feasibility study are to:

i) ascertain the acceptability of a randomised control trial for women with PND;

ii) explore effective methods of recruitment;

iii) explore the participants' and health professionals' views about the intervention;

iv) ascertain the acceptability of the outcome measures;

v) identify a cost-effectiveness measure;

vi) provide an estimate of the size of change that might be expected with such an intervention to inform the power calculation for the larger RCT;

vii) build a working alliance with health care professionals for the larger clinical trial;

viii) make any necessary adaptations to the intervention and develop a full proposal for a main RCT to be submitted to the MRC in 2012.

## Methods/Design

The study design adopts the MRC framework for the development and evaluation of complex interventions [28]. Ethical approval for the pilot and feasibility trial was obtained from Coventry and Warwickshire Research Ethics Committee (ID number 08/H1211/94).

### Peer-supporters

Health visitors in Coventry and Warwickshire PCTs identified potential participants to be trained as peer-supporters (n = 18). They were recruited by personal invitation using a specification that set out essential and desirable attributes established from stakeholder consultation, including that they had a) recently experienced PND (i.e. within the last five years) b) fully recovered from depression; c) an empathic and non-judgmental disposition; and d) could commit the time to participate in the training and provide the telephone support. Multiple assessments of mental health and social wellbeing were made and their GPs were required to confirm the suitability of individuals identified for the proposed peer-support role. Eligible peer-supporters were invited to attend a training programme lasting approximately eight hours, to develop their understanding of the role of the peer-supporter and their confidence to deliver the intervention. The training was based on Dennis's training manual [14], but was adapted to include other material about active listening skills and promoting successful behaviour change [29,30], encouraging goal-setting and decision-making [31]. The training was provided locally, and crèche facilities were made available.

### Inclusion criteria

#### Inclusion criteria

women >16 years of age at the time of giving birth and who are experiencing depressive symptomatology (i.e. EPDS > = 10 and/or clinical judgment) at or after the 8-week check, and who are potentially receptive to receiving telephone support.

#### Exclusion criteria

women with a score of 23 or above on the EPDS; women who pose a suicide risk or a risk to their children; women receiving specialist psychiatric care or experiencing any mental illnesses (other than PND) or learning difficulties, or who are not able to speak English, or who are not accessible via the telephone. Participation in the study is only undertaken with the consent of the participant, their health visitor, and their GP.

### Recruitment

All health visitors within Coventry and Warwickshire Care Trusts (PCTs) are recruiting to the study. Potential participants are screened for eligibility by the health visitors at the 8-week postnatal check using either the Edinburgh Postnatal Depression Scale (EPDS > = 10) or the three Whooley questions recommended by NICE [13]. Eligible women are then given a brief information leaflet about the study, and women who would like further information are asked for their written consent for the health visitor to give their contact details to the research team.

In addition to the recruitment method outlined above, the study information leaflet is available within GP surgeries and Children's Centres within Coventry and Warwickshire allowing participants to self-refer into the study by contacting the research team directly. Participants can also access information about the study using the link at 'Netmums' or via the Warwick University webpage. Eligible patients are also being referred to the study from the 'Improved Access to Psychological Therapies (IAPT) waiting-list.

Following referral to the study team, a researcher sends a full information sheet and contact with the mother is arranged two weeks later, to discuss her participation. Once a participant has agreed to take part and provided consent, their details are passed onto the Clinical Trials Unit at the University of Warwick for randomisation. The group allocation information is provided to the researcher who then informs the participant.

### Intervention group

All participants receive standard care from their GP and health visitor. Women allocated to the intervention group also receive telephone support calls over a period of 4 months from peer-supporters who have been specially trained to deliver the intervention (i.e. the same peer-supporters that delivered the pilot study intervention). Outcome measures tested in the pilot phase are being collected at baseline, 2- and 4-months.

### Sample Size

A total of 30 participants are being recruited to study. This will enable us to detect an effect size of around 0.6sd using a power of 80% and two-sided significant level of 95%. Analysis of the data will be carried out on an intention-to-treat basis.

### Outcome Measures

The primary outcome measure is depressive symptomology, which is being measured using the Edinburgh Postnatal Depression Scale [32]. Secondary outcome measures to assess maternal functioning include: Hospital Anxiety and Depression Scale [33], Parenting Sense of Competence Scale [34], Dyadic Adjustment Scale [35], Emotional Support Questionnaire [36] and Self-Efficacy [37]. The Care Index [38] is being used to assess the interaction between the mother and baby. Maternal perceptions of the telephone peer-support and are being assessed using the Peer-support Evaluation Inventory.

### Process Data

In-depth, semi-structured interviews will be conducted with a random sample of all stakeholders to establish the acceptability and feasibility of the intervention for participants, peer-supporters and health visitors.

### Cost-Effectiveness

A prospective economic evaluation is being conducted. The focus is on the additional costs of delivering the training programme for the services involved. The costs of training will be calculated using a record of the resources employed. Unit costs for service delivery will be taken from a national compendium (e.g. costs of training and supervision) and multiplied by the intensity of the service used by each family. A 'Service Use' Questionnaire is being used to collect data on public service utilisation by study participants. Unit costs will be obtained from national databases. Training, delivery and service utilisation costs will be combined to provide an estimate of the total health and social care cost in each arm. The expected incremental cost-effectiveness ratio for peer-support vs. usual care in the prevention of PND cases will be estimated. A within-trial probabilistic sensitivity analysis will be undertaken using non-parametric bootstrap method. The results will be presented as ICERs and cost- effectiveness acceptability curves. Scenario analyses will be used to examine the impact of differential training and resourcing models on the expected cost-effectiveness. The data collected in the trial will be used to inform pre-trial modelling as part of the design process for a future full scale RCT with economic evaluation.

### Data Analyses

#### i) *Quantitative*

Descriptive methods will be used to describe participant characteristics, to compare the 'refusers' with the study participants, and to report levels of participation and drop out. Comparison of intervention and control group outcome data will be provided with regard to the outcome measures described above. The results of the statistical analyses will be used to reach some preliminary conclusions regarding the viability and acceptability of the intervention, the usefulness of the outcome measures being used, and the sample size required in a full trial.

#### ii) *Qualitative*

Tape-recorded semi-structured interviews will be transcribed verbatim. A thematic framework approach [39] will be used to generate themes from the transcripts.

The embedded mixed-methods design of the study will enable the quantitative and qualitative data to be analysed iteratively.

## Discussion

A reduction in a woman's depressive symptomatology could potentially produce an improved mother-infant relationship in affected dyads, with significant consequences in terms of the capacity of the infant for affect regulation. This could also impact on the later emotional and behavioural adjustment of the child, especially in the case of disadvantaged boys. Poor emotional and behavioural adjustment in the early years is associated with a range of poor long-term outcomes including delinquency, drug abuse, and a range of mental health and relationship problems, which are very costly for NHS and other services. This form of provision could therefore have an immediate impact on health service use and in the long-term improve a range of public health outcomes about which there is currently considerable concern, and to which postnatal depression undoubtedly makes a significant contribution.

The proposed study will develop current innovative work in peer-led support interventions and telecare by applying existing expertise to a new domain (i.e. PND), testing the feasibility of a peer-led telephone intervention for mothers living with PND, and developing the relationship between the lay and clinical communities. The outcome of the proposed study will potentially benefit a significant number of patients and support a future application for a larger study to undertake a full evaluation of the clinical and cost effectiveness of the intervention.

## Abbreviations

PND: Postnatal depression; MRC Framework: Medical Research Council; EPDS: Edinburgh Postnatal Depression Score; NICE: National Institute for Health and Clinical Excellence; DSM IV: Diagnostic and Statistical Manual of Mental Disorders Fourth Edition; APA: American Psychiatric Association; NHS: National Health Service; DoH: Department of Health; PCT: Primary Care Trust; IAPT: Improved Access to Psychological Services; ICERs: Incremental Cost Effectiveness Ratios; RCT: Randomised Control Trial;

## Competing interests

The authors declare that they have no competing interests.

## Authors' contributions

JB and IC undertook the development and setting up of the study and SS took on the role of the researcher in the second stage (pilot study). KMc trained and supervises all the peer-supporters. All authors have read and approved the final version of this manuscript.
